# Supplementary material for: Exploring contextual effects of post-migration housing environment on mental health of asylum seekers and refugees: A cross-sectional, population-based, multi-level analysis in a German federal state
Source: PLOS Glob Public Health. 2023 Dec 27;3(12):e0001755. doi: 10.1371/journal.pgph.0001755 (PMC10752521; doi:10.1371/journal.pgph.0001755)
Supplement: Attachment — Submitted filename: Response to reviewers.docx [file pgph.0001755.s001.docx]

**Response to editorial and reviewers’ requests**

Journal requirements:

1. Please amend your detailed Financial Disclosure statement. This is published with the article. It must therefore be completed in full sentences and contain the exact wording you wish to be published.

a. Please clarify all sources of funding (financial or material support) for your study. List the grants (with grant number) or organizations (with url) that supported your study, including funding received from your institution. 
b. State the initials, alongside each funding source, of each author to receive each grant.
c. State what role the funders took in the study. If the funders had no role in your study, please state: “The funders had no role in study design, data collection and analysis, decision to publish, or preparation of the manuscript.”
d. If any authors received a salary from any of your funders, please state which authors and which funders.

We have added the information by which authors the funding was received. All other requirements have already been met in the manuscript.

2. Since your data is not available for proprietary reasons, please explain via email why the data is not available. Please also include the contact information for the third party organization that should be contacted should other researchers want to request access to this data and please include the full citation of where the data can be found. We also request that you verify with us via email that any researcher will be able to obtain the data set in the same manner that the you have obtained it. If you feel you are unwilling or unable to adhere to this policy, please explain your reasons by return email and your exemption request will be escalated to the editor for approval. Your exemption request will be handled independently and will not hold up the peer review process, but will need to be resolved should your manuscript be accepted for publication. One of the Editorial team will be in touch if they require more information.

The statement has been amended.

Reviewer 1:

The evaluated manuscript aimed to analyze contextual effects of post-migration housing environment on their mental health.
Congratulations to the authors because I consider that it is an interesting manuscript and of great public health relevance, however I must make the following observations and suggestions:

We thank the reviewer for their time and effort put forward to ensure high-quality research. In the following, we respond to all points raised one by one.

1. In the abstract, the authors list the results of the ORs that the model yielded, however none of these show a statistically significant result. I consider that it is useless to place these values and it would be more appropriate to describe the results of the variables corresponding to the second level of analysis and finally mentioning that none had effects on the mental health of the population analyzed in this study.

We have made appropriate changes to the abstract and followed the reviewer’s recommendation.

2. What does it mean for the authors that a tendencies were found? I suggest avoiding this phrase in the presence of statistical results without significance. It is prudent to point out that the frequencies and proportions are higher in a group with depression or anxiety, but the word “tendencies” is ambiguous.

We have followed the reviewer’s recommendation and dropped the term ‘tendencies’. Originally, we used the term to describe clearly higher odds in the ‘exposed’ group, corresponding with the literature and our expectation, while lacking statistical significance, potentially due to low statistical power.

3. The methods are appropriately described and the use of multilevel analysis is adequately presented, some doubts about the variables that characterize each level arise when reading them are
a. Why was a sample size not calculated?
b. Why were the centers dichotomized into ≥50 people per center? Could you explain what is the usual population that exists in these centers on average?
c. The region of origin definition that is analyzed is very broad and perhaps does not reflect the factor that could really be influencing, for example, coming from a low-income country or at war.
d. Why was 20 minutes considered as the time to calculate the remoteness index? What is the optimal time for these transfers in Germany?

a) A-priori sample size calculation was not possible given that the individual-level health data was collected prior to the conceptualization and setup of the contextual-level data. Furthermore, the data analysis was explorative, and common recommendations speak against sample size calculation for post-hoc or exploratory analyses.

b + d) The decision to dichotomize the size of each accommodation at 50 residents and remoteness at 20minutes, was made to keep our statistical model lean and less prone to statistical error. By reducing the number of categories, we aimed to reduce the risk for incorrect deductions and comply with our small sample size. The cut-offs were chosen after thorough discussions in the team and based on various factors. For the number of residents, there is no “usual population” as the range is very broad. Cut-off at 50 residents was close to the median based on the number of residents (see table 2) and was deemed a suitable cut-off by the departmental team. The remoteness cut-off was set to 20 minutes as it is the time period which the German Association of Statutory Health Insurance Physicians (*Kassenärztliche Vereinigung*) considers acceptable and uses for their own ambulatory health care planning. Appropriate changes to elaborate on this have been made in the manuscript’s methodology.

c) The data on region of origin is available on the level of nationalities. Given the small sample size and power restrictions, we decided to aggregate the participants on the level of region of origin.

4. Regarding the results, in Table 3 "Sample description", where the frequencies and proportions for the variables of each level are described, the number of participants do not coincide with the total sample size shown in Table 2

The difference in sample size is due to missing data. Each case with any missing data was excluded from each calculation (listwise deletion), leading to a smaller sample in the generalized models compared to the descriptive analyses.

5. The titles of the tables do not adequately inform their content

Further details have been added to the tables’ titles.

6. Figures must have their own caption containing the explanation found in the text.

We have added captions for figures 1 and 2.

7. The discussion of this work is very limited and I consider it its greatest weakness, the authors do not compare their results with those found in other populations, they do not consider that in reality the post-migration housing environment has no effect on depression or anxiety and what would be the possible explanations for this in this population of Germany.

We have re-worked the section on discussion, to better contrast and critique our findings in the context of other research.

8.The conclusion must be rewritten because it is ambiguous and the results of the analysis are clear.

We have re-worked the section on discussion, to better contrast and critique our findings in the context of other research.

Reviewer 2

The authors abide to the truth and are honest about the absence of significant differences, which should be praised. However, and due to its limited findings, this manuscript should be submitted only as a short communication, which could be useful to other scientists in the designing of similar studies.

We thank the reviewer for their praise and honest feedback. We believe that a full manuscript explaining the premises behind the research conducted, its detailed methodology and analyses leading to the discussion, offers the highest value for readers, including those aiming to design similar studies. Furthermore, non-significant results are results as well and we believe a detailed elaboration of methods and results will support future studies, and eventually be beneficial for systematic reviews or studies trying to synthesis effects and effects sizes which then should equally well include non-significant results to avoid publication bias.


Reviewer 3

This original article explored the effect of post-migration on anxiety and depression symptoms (and other sociodemographic aspects) in asylum seekers and refugees in a German federal state. The proposed results show a clear need for improvement of well-being of this population. Some recommendations are presented below.

We thank the reviewer for their time and effort put forward to ensure high-quality research. In the following, we respond to all points raised one by one.

-If possible, the symptoms of anxiety and depression (GAD-2, PHQ-2) could be further explored if descriptive data are presented on each item analyzed in the study participants.

Given the validation of the screening instruments was conducted with the 2-item questionnaires in terms of specificity and sensitivity, we decided to focus the analyses on the fully constructed instrument, not individual symptoms.

-It is required not to generalize to "mental health" only with the presence of anxiety and depression. In line 120 it could be clarified in a more detailed way.

We have amended the manuscript to reflect our focus on symptoms of depression and generalized anxiety.

-I recommend adding a univariate analysis of Table 2, to identify the associations between the presented variables .

As noted in the manuscript, we have abstained from conducting such univariate analyses to prevent a ‘Table 2 fallacy’ (see manuscript references). Given our study conceptualization focusing on the reported mental health outcomes, calculating and reporting associations in table 2 may encourage false conclusions which have not carefully considered confounding. Additionally, the selection of variables for fitting our main statistical models was based on theory and not any purely statistical decision-making.

-The discussion section requires further analysis of all the variables analyzed. It is required that the results obtained be hypothesized and compared with those of other authors.

We have re-worked the section on discussion, to better contrast and critique our findings in the context of other research.

-I recommend reducing the extension of the analysis of the generalized linear models, since most of the results did not have statistical significance and/or the confidence intervals presented do not have enough statistical power to take them as valid.

Our planned and conducted statistical analyses leading to statistically non-significant results are part of our findings and may inform researchers in future studies. For the sake of completeness and full transparency, we have decided to include the analyses and their findings. We discuss the limitations and likely role of statistical power, omitting the detailed estimates would in our view be not transparent.

-Place the corresponding abbreviations at the foot of tables 2 and 3.

We have added all abbreviations at the foot of all tables.
