# Supplementary material for: Exploring contextual effects of post-migration housing environment on mental health of asylum seekers and refugees: A cross-sectional, population-based, multi-level analysis in a German federal state
Source: PLOS Glob Public Health. 2023 Dec 27;3(12):e0001755. doi: 10.1371/journal.pgph.0001755 (PMC10752521; doi:10.1371/journal.pgph.0001755)
Supplement: Attachment — Submitted filename: Response to reviewers.docx [file pgph.0001755.s002.docx]

**Response to editorial and reviewers’ requests**

Many thanks to both the editor and the reviewers for their time and effort in ensuring a high-quality publication.

In the following, we are addressing each reviewer remark individually in blue font color:

1. If the authors have adequately addressed your comments raised in a previous round of review and you feel that this manuscript is now acceptable for publication, you may indicate that here to bypass the “Comments to the Author” section, enter your conflict of interest statement in the “Confidential to Editor” section, and submit your "Accept" recommendation.

 Reviewer #1: All comments have been addressed

 Reviewer #2: All comments have been addressed

 Reviewer #4: (No Response)

2. Does this manuscript meet PLOS Global Public Health’s [publication criteria](https://journals.plos.org/globalpublichealth/s/criteria-for-publication)? Is the manuscript technically sound, and do the data support the conclusions? The manuscript must describe methodologically and ethically rigorous research with conclusions that are appropriately drawn based on the data presented.

 Reviewer #1: Yes

 Reviewer #2: Yes

 Reviewer #4: Yes

3. Has the statistical analysis been performed appropriately and rigorously?

 Reviewer #1: Yes

 Reviewer #2: Yes

 Reviewer #4: Yes

4. Have the authors made all data underlying the findings in their manuscript fully available (please refer to the Data Availability Statement at the start of the manuscript PDF file)?

The [PLOS Data policy](https://journals.plos.org/globalpublichealth/s/data-availability) requires authors to make all data underlying the findings described in their manuscript fully available without restriction, with rare exception. The data should be provided as part of the manuscript or its supporting information, or deposited to a public repository. For example, in addition to summary statistics, the data points behind means, medians and variance measures should be available. If there are restrictions on publicly sharing data—e.g. participant privacy or use of data from a third party—those must be specified.

 Reviewer #1: Yes

 Reviewer #2: Yes

 Reviewer #4: No

5. Is the manuscript presented in an intelligible fashion and written in standard English?

PLOS Global Public Health does not copyedit accepted manuscripts, so the language in submitted articles must be clear, correct, and unambiguous. Any typographical or grammatical errors should be corrected at revision, so please note any specific errors here.

Reviewer #1: Yes

Reviewer #2: Yes

Reviewer #4: Yes

6. Review Comments to the Author

Please use the space provided to explain your answers to the questions above. You may also include additional comments for the author, including concerns about dual publication, research ethics, or publication ethics. (Please upload your review as an attachment if it exceeds 20,000 characters)

Reviewer #1: The authors have adequately responded to the comments, I consider that now the manuscript can be published in the present form

Thank you for your contribution.

Reviewer #2: I find very intriguing the finding about a "protective" effect of deprivation before depression; however, despite the study recognizes its limitations, its findings warrant future studies to determine if the tendencies found are consistent among ASR, not only in Germany, but in other countries.

Thank you for this suggestion. We have now included a paragraph on this (lines 360-365).

Reviewer #4: Dear Authors,

Thanks for sharing this manuscript. I enjoyed reading it.

Your manuscript provides an important overview of the effects of the post-migration housing environment on the mental health of asylum seekers and refugees in Germany. Housing situation is a determinant of health that is underreported in the context of migrant health. The study has many merits; however, I recommend the authors address the following points in their manuscript:

Methods:
- The authors referred to previously reported details about "Setting, sampling and recruitment". However, they should provide more details about their study’s setting and data collection. This should include the data collection period, the languages into which the questionnaire was translated, and the rationale for choosing these languages.

We have added details on data collection period, language of questionnaire, and the rationale for this (lines 115-118).

- Did the authors involve the concerned population (asylum seekers and refugees) in the design and implementation of their study? If not, why? This should be reflected in the limitations.

Asyum seekers were involved in the design of the questionnaire by means of pre-testing selected instruments and incorporating their feedback on comprehensibility and linguistic diversity.

Furthermore, the design was informed by preceding extensive qualitative studies on living situations in accommodation centers which gave voice to asylum seekers regarding their social determinants of health and the related challenges in camps and shared accommodation facilities. The insights from these emic perspectives informed the focus on accommodation centers.

A paragraph has been added to the manuscript (see lines 119-124) and references for further information cited.

- The length of stay in the collective accommodation centres is a potential confounder that has yet to be considered in the analyses. Authors should explain why they did not include this confounder and reflect on this in the limitations.

We have added details on the lack of data on length of stay in the limitations section (lines323-324).

Discussion:
- How would this study’s outcomes apply to other parts of Germany?

As discussed in the manuscript, the analyses utilize a quasi-random allocation of ASR to the German states and the districts within them, and allows for improved un-confoundedness, and potentially generalizability.

- Could the authors put their study in the context of the current situation of asylum seekers and refugees in Germany, considering the high number of people who fled Ukraine to Germany?

A direct comparison or contrasting of the different ASR populations is out of scope for this manuscript. The current Ukrainian refugees arrive(d) in Germany with a protected status as war refugees and do not need to apply for asylum in Germany. As such they are not part of the usual trajectory in terms of housing, are not placed in the initial state-wide reception centres, and have high residential mobility.

Best wishes for you resubmission.

We thank the author for their contribution and hope our manuscript can offer the highest value to the readers interested in contextual effects of housing environment on mental health of ASR.
